# Intra-Genomic Heterogeneity in 16S rRNA Genes in Strictly Anaerobic Clinical Isolates from Periodontal Abscesses

**DOI:** 10.1371/journal.pone.0130265

**Published:** 2015-06-23

**Authors:** Jiazhen Chen, Xinyu Miao, Meng Xu, Junlin He, Yi Xie, Xingwen Wu, Gang Chen, Liying Yu, Wenhong Zhang

**Affiliations:** 1 Department of Infectious Diseases, Huashan Hospital, Fudan University, Shanghai 200040, China; 2 Department of Stomatology, Huashan Hospital, Fudan University, Shanghai 200040, China; The University of Hong Kong, HONG KONG

## Abstract

**Background:**

Members of the genera *Prevotella*, *Veillonella* and *Fusobacterium* are the predominant culturable obligate anaerobic bacteria isolated from periodontal abscesses. When determining the cumulative number of clinical anaerobic isolates from periodontal abscesses, ambiguous or overlapping signals were frequently encountered in 16S rRNA gene sequencing chromatograms, resulting in ambiguous identifications. With the exception of the genus *Veillonella*, the high intra-chromosomal heterogeneity of *rrs* genes has not been reported.

**Methods:**

The 16S rRNA genes of 138 clinical, strictly anaerobic isolates and one reference strain were directly sequenced, and the chromatograms were carefully examined. Gene cloning was performed for 22 typical isolates with doublet sequencing signals for the 16S rRNA genes, and four copies of the *rrs-ITS* genes of 9 *Prevotella intermedia* isolates were separately amplified by PCR, sequenced and compared. Five conserved housekeeping genes, *hsp60*, *recA*, *dnaJ*, *gyrB1* and *rpoB* from 89 clinical isolates of *Prevotella* were also amplified by PCR and sequenced for identification and phylogenetic analysis along with 18 *Prevotella* reference strains.

**Results:**

Heterogeneity of 16S rRNA genes was apparent in clinical, strictly anaerobic oral bacteria, particularly in the genera *Prevotella* and *Veillonella*. One hundred out of 138 anaerobic strains (72%) had intragenomic nucleotide polymorphisms (SNPs) in multiple locations, and 13 strains (9.4%) had intragenomic insertions or deletions in the 16S rRNA gene. In the genera *Prevotella* and *Veillonella*, 75% (67/89) and 100% (19/19) of the strains had SNPs in the 16S rRNA gene, respectively. Gene cloning and separate amplifications of four copies of the *rrs-ITS* genes confirmed that 2 to 4 heterogeneous 16S rRNA copies existed.

**Conclusion:**

Sequence alignment of five housekeeping genes revealed that intra-species nucleotide similarities were very high in the genera *Prevotella*, ranging from 94.3–100%. However, the inter-species similarities were relatively low, ranging from 68.7–97.9%. The housekeeping genes *rpoB* and *gyrB1* were demonstrated to be alternative classification markers to the species level based on intra- and inter-species comparisons, whereas based on phylogenetic tree *rpoB* proved to be reliable phylogenetic marker for the genus *Prevotella*.

## Introduction

Periodontal abscess is a suppurative lesion that is associated with periodontal breakdown and localised pus in the gingival wall of the periodontal pocket. Gram-negative, strictly anaerobic bacteria and partially facultative anaerobes are the predominant microorganisms that cause periodontal disease in humans [1]. Previously, we reported that the predominant culturable obligate anaerobic pathogens isolated from periodontal abscesses are members of the genera *Prevotella*, *Veillonella* and *Fusobacterium* [2]. These genera also comprise a portion of the indigenous microbiota of the human and animal gastrointestinal tract and oral cavity [2, 3]. In addition to dental diseases, they play a role in extraoral infections, such as cellulitis, intra-abdominal, urogenital and osteoarticular infections and bacteraemia [4–8].

Because a large number of novel species or anaerobic genera have been isolated, proposed or reclassified as Gram-negative anaerobic rods, the taxonomy has changed significantly in the recent past [9–12]. For *Prevotella* in particular, additional in-depth studies focused on the gut microbiome and oral diseases have led to the recent identification of large numbers of novel species [13–17]. The comparison of 16S rRNA genes (*rrs*) by RFLP or sequencing is widely performed to estimate the evolutionary history, provide taxonomic classification and identify clinical isolates [18–23]. Although the *rrs* gene is accepted as the gold standard molecular clock, use of the *rrs* gene has been challenged by the diversity of multiple heterogeneous copies and the low resolution of closely related species [24].

When identifying the cumulative number of anaerobic strains in our studies, we have frequently encountered ambiguous or overlapping signals in *rrs* gene sequencing chromatograms, even with repeated single clone isolation and sequencing. The most reasonable explanation for these results is the heterogeneity of multiple *rrs* genes. The high intra-chromosomal heterogeneity of *rrs* genes has been reported for the genus *Veillonella* [19, 25], but no such phenomena have been reported in other clinically relevant anaerobic bacteria. In contrast, in genome databases, all four copies of *rrs* in *Prevotella* type strains are identical, including those of *P*. *intermedia* 17, *Prevotella melaninogenica* ATCC 25845, *Prevotella denticola* F0289 and *Prevotella dentalis* DSM 3688.

To investigate this discrepancy in more detail, we selected 138 clinical anaerobic strains isolated from periodontal abscesses to determine whether they contained multiple heterogeneous copies of *rrs* and to assess the extent of intra-genomic variation. In addition, to improve the identification and phylogenetic classification of clinical *Prevotella* isolates, we evaluated the suitability of five conserved genes, *rpoB*, *dnaJ*, *recA*, *hsp60* and *gyrB*, as alternative identification markers and molecular clocks for *Prevotella* in 89 clinical isolates and 18 reference species from a genomic database. Conserved housekeeping genes, such as *rpoB* and *hsp60*, are more discriminative than the *rrs* gene and have been suggested as possible molecular clocks for bacterial phylogenetic studies [26, 27]. Other genes such as *gyrB*, *recA* and *dnaJ* provide additional information that supplement 16S rRNA gene sequence analysis and have also been suggested for phylogenetic studies and multilocus sequence analysis [27–31].

## Materials and Methods

### Clinical anaerobic strains and reference strains

Patients who suffered from periodontal abscesses routinely undertook anaerobic bacterial culture examination and antimicrobial susceptibility tests at the Department of Stomatology of Huashan Hospital (Shanghai, China). Isolation, culturing methods and partial description of the distribution of 100 strains were previously described [2]. In detail, the abscesses were drained after decontamination of the mucosa. A sterile inoculating loop was inserted into the deep area of the fistula for 20 seconds. The loop was then immediately inoculated onto pre-reduced culture medium, specifically Anaerobe Basal Agar (Oxoid, Oxoid Ltd, UK) plates supplemented with 5% sterile defibrinated sheep blood, using quadrate section streak methods. The culture medium was immediately incubated in GENbags (bioMérieux, France) at 37°C for 2–4 days of growth. Typical anaerobic colonies with a distinct morphology were selected, cultured and preserved in our laboratory for use in oxygen tolerance tests and antimicrobial susceptibility tests. Informed written consent was obtained from each patient. The present study is approved by the Ethics Committee from Huashan Hospital, Fudan University.

A total of 138 clinical, strictly anaerobic isolates preserved in the laboratory were re-inoculated and cultured. Each strain was purified by sub-culturing a single colony.

Genome sequences of 18 reference or type strains were obtained from the GenBank database (http://www.ncbi.nlm.nih.gov/genome/). The eighteen strains included *P*. *intermedia* 17, *Prevotella melaninogenica* ATCC 25845, *Prevotella melaninogenica* D18, *Prevotella nigrescens* ATCC 33563, *Prevotella pallens* ATCC 700821, *Prevotella histicola* F0411, *Prevotella multiformis* DSM 16608, *Prevotella denticola* F0289, *Prevotella denticola* CRIS 18C-A, *Prevotella veroralis* F0319, *Prevotella oris* F0302, *Prevotella salivae* DSM 15606, *Prevotella dentalis* DSM 3688, *Prevotella buccae* ATCC 33574, *Prevotella buccae* D17, *Prevotella oulorum* F0390, *Candidatus Prevotella conceptionensis* 9403948, *Alloprevotella rava* F0323 and *Bacteroides fragilis* 638R (out-group species).

If the genome had not been annotated, housekeeping genes were predicted from genome sequences using homologous genes of the most similar species identified by BLASTN. In most *Prevotella* type strains, two similar but distinct homologues of *gyrB* were identified using BLASTN, and the annotation was not uniform in previous reports. In our study, *gyrB1* was defined as the higher-similarity homologue to the only *gyrB* gene of *B*. *fragilis* 638R. The GenBank/EMBL/DDBJ accession numbers of the sequences studied are listed in S2 Table.

### Identification of the clinical strains and analysis of the intragenomic diversity of *rrs* genes

Genomic DNA from all isolates was extracted as previously described [2]. The *rrs* gene was amplified to almost full length using the universal primers 8FLP (5’-AGTTTGATCCTGGCTCAG-3’) and 1492RPL (5’-GGTTACTTGTTACGACTT-3’) or 1527R (5’-AGAAAGGAGGTGATCCAGCC-3’) with Premix *Ex-Taq* enzyme (Takara, Japan). For some isolates, if the 1527R primer yielded weak or no PCR product, the primer 1492R was used instead. The PCR with Premix *Ex-Taq* enzyme was performed using the following program: 95°C for 5 min; followed by 35 cycles consisting of 94°C for 30 s, 55°C for 30 s and 72°C for 2 min; with a final extension period at 72°C for 5 min. The amplification products were detected using electrophoresis and sequenced using a 3730xl DNA Analyzer (Applied Biosystems, USA) by the same amplification primers. Sequence chromatograms were carefully examined, and the numbers of overlapping nucleotides and positions of base loci were recorded.

In order to confirm whether the sequence result was enzyme related or not, the *rrs* gene of 8 isolates, *P*. *denticola* M04, *P*. *buccae* M27-2, *P*. *intermedia* M57-2, *P*. *buccae* M70, *P*. *melaninogenica* M71, *P*. *intermedia* M83, *P*. *oris* Y04, *P*. *denticola* Y78, and one reference strain *P*. *melaninogenica* ATCC 25845 was also amplified with Q5 high-fidelity polymerase (New England Biolabs, UK) and compared with the amplification by the Premix *Ex-Taq* enzyme. The PCR with Q5 enzyme was performed using the following program: 98°C for 2 min; followed by 35 cycles consisting of 98°C for 15 s, 55°C for 15 s and 72°C for 1 min; with a final extension period at 72°C for 5 min.

Clean *rrs* gene sequences from 16S ribosomal RNA sequences (Bacteria and Archaea) and the Nucleotide collection (nr/nt) databases were analysed using the BLASTN program. Identification at the species and genus level was defined at *rrs* sequence similarities of >99% and >97%, respectively, with the prototype strain sequences in the databases [32]. For isolates with ambiguous signals in direct sequencing, *rrs* sequences were obtained from gene cloning as described below.

### *rrs* gene cloning

To verify the *rrs* gene heterogeneity observed in direct sequence chromatograms, characterised by overlapping signals only in or from certain positions of the gene, *rrs* gene cloning was performed in 15 typical clinical *Prevotella* isolates that had the greatest number of overlapping chromatogram signals and 13 unidentifiable isolates (Table 1) that had mixed *rrs* sequencing signals. The *rrs* genes were re-amplified using Q5 high-fidelity polymerase (New England Biolabs, UK) by 8FLP, 1492RPL and 1527R primers under conditions described above, purified by QIAquick PCR Purification kit (Qiagen, Germany) according to the manufacturer’s instructions, inserted into a cloning vector and transformed into *E*. *coli* DH5α competent cells using the pEASY-Blunt Zero Cloning Kit (Transgen Biotech, China) according to the manufacturer’s instructions. For each strain, clones between 8 to 14 were isolated and sequenced. *Alloprevotella rava* was previously classified as *Prevotella oral taxon 302*. Although it has been separated from the genus *Prevotella*, this strain was analysed together with the other *Prevotella spp*. in this study. Sequences from each clone were compared with the direct sequencing chromatograms of the isolates. Those clones that had identical *rrs* sequences were counted and defined as one copy. We used copy A, B, C and D to designate different copies of *rrs* genes and ranked by numbers of clones.

**Table 1:** 16S rRNA gene cloning of 22 *Prevotella spp*. clinical isolates and four copies of *rrs* sequencing of 9 *Prev*otella intermedia *clinical* isolates.

	Specific colonies/total colonies			
Strains	Copy A	Copy B	Copy C	Copy D
*P*. *intermedia* HJX081-2	6/9	3/9	-	-
*P*. *intermedia* HJM050	6/10	4/10	-	-
*P*. *intermedia* HJM056	6/9	3/9	-	-
*P*. *intermedia* HJM057-2	4/8	3/8	1/8	-
*P*. *intermedia* HJM069	5/11	4/11	2/11	-
*P*. *intermedia* HJM061	5/8	2/8	1/8	-
*P*. *intermedia* HJH14	4/8	4/8	-	-
*P*. *intermedia* HJH16	5/8	3/8	-	-
*P*. *buccae* HJM027-2	5/8	2/8	1/8	-
*P*. *buccae* HJM070	4/8	3/8	1/8	-
*P*. *denticola* HJX050	6/8	1/8	1/8	-
*P*. *denticola* HJX078	4/8	3/8	1/8	-
*P*. *melaninogenica* HJH25	8/14	3/14	3/14	-
*P*. *nigrescens* HJH18	4/8	2/8	1/8	1/8
*P*. *nigrescens* HJX026	4/8	3/8	1/8	-
*P*. *nigrescens* HJX052-2	8/12	4/12	-	-
*P*. *nigrescens* HJM025	5/8	3/8	-	-
*P*. *nigrescens* HJM032	7/11	4/11	-	-
*P*. *nigrescens* HJM066	5/8	3/8	-	-
*P*. *nigrescens* HJX046	4/8	4/8	-	-
*P*. *oris* HJH23	3/8	2/8	2/8	1/8
*Alloprevotella rava* HJM063	3/8	2/8	2/8	1/8
**Four copies of *rrs***	Copy A	Copy B	Copy C	
*P*. *intermedia* HJX081-2	*rrs-1*, *rrs-2*	*rrs-3*, *rrs-4*	-	-
*P*. *intermedia* HJM050	*rrs-1*, *rrs-3*	*rrs-2*, *rrs-4*	-	-
*P*. *intermedia* HJM056	*rrs-2*, *rrs-4*	*rrs-1*, *rrs-3*	-	-
*P*. *intermedia* HJM057-2	*rrs-2*, *rrs-3*	*rrs1*	*rrn-4*	-
*P*. *intermedia* HJM069	*rrs-4*	*rrs-2*	*rrs-1*	*rrs-3*
*P*. *intermedia* HJX070	*rrs-1*, *rrs-3*, *rrs-4*	*rrs-2*	-	-
*P*. *intermedia* HJX057	*rrs-2*, *rrs-3*	*rrs1*	*rrn-4*	-
*P*. *intermedia* HJM034	*rrs-1*, *rrs-4*	*rrs-2*, *rrs-3*	-	-
*P*. *intermedia* HJX080	*rrs-1*, *rrs-2*	*rrs-3*, *rrs-4*	-	-

### Sequencing four copies of *rrs-ITS* genes of *P*. *intermedia*

*P*. *intermedia* has 4 copies of the *rrs-ITS* genes in its genome, which are highly similar. However, the flanking regions of the 4 genes are distinct. Four primers, Inter_rrn1-F, Inter_rrn2-F, Inter_rrn3-F and Inter_rrn4-F (S1 Table) specifically targeting the flanking regions of the four copies of the *rrs* sequences and one universal primer 23S_1-60-R (S1 Table) targeting the *rrs*-ITS region were designed based on the genome sequence of *P*. *intermedia* 17. Flanking region primers instead of universal primers can amplify each *rrs*-ITS genes separately and verify the intragenomic heterogeneity. Four copies of *rrs*-ITS genes were therefore amplified with Premix *Ex-Taq* enzyme (Takara, Japan) using Inter_rrn1-F, Inter_rrn2-F, Inter_rrn3-F, Inter_rrn4-F and 23S_1-60-R primers and the conditions are listed in S1 Table. Amplicons of approximately 2100 bp covering the entire length of the *rrs* and ITS regions in nine *P*. *intermedia* clinical isolates with typical overlapping chromatogram signals were sequenced. Four copies of *rrs* sequences were compared with the results of the direct sequencing chromatograms. For isolates which had cloning results, each *rrs*-ITS sequence was matched to copy A/B/C/D sequences from cloning and then a designation to copy A/B/C/D was made. For isolates which had no cloning results, copy A, B, C and D were used to designate different copies of *rrs* genes and ranked by copy numbers.

### Conserved gene sequences and phylogenetic analysis

Five conserved housekeeping genes and the 16S rRNA gene in members of the *Prevotella* genus were selected for identification and phylogenetic analysis. A partial *rpoB* gene (approximately 2000 bp), *hsp60* (approximately 600 bp), *recA* (approximately 650 bp), *dnaJ* (approximately 850 bp) and *gyrB1* (approximately 1250 bp) were amplified from 89 clinical isolates of *Prevotella* using the degenerate primers and conditions listed in S1 Table. PCR was also performed with Premix *Ex-Taq* enzyme (Takara, Japan) and the conditions are listed in S1 Table. The amplicons were detected using electrophoresis in agarose gels (1.2% w/w) and sequenced using sequencing primers (S1 Table) with a 3730xl DNA Analyzer.

Consensus sequences for all genes and clones were assembled using Lasergene SeqMan II software (DNAStar, Inc., Madison, WI) with default parameters. For 16S rRNA genes that had indels in direct sequencing, the sequence of one clone of the gene was used for alignment and phylogenetic tree construction. Five conserved housekeeping genes and an approximately 1320-bp length of *rrs* (nucleotides 106–1421 based on the *E*. *coli rrs* numbering system) were aligned in MEGA5 using CLUSTAL W and corrected by manual inspection. A phylogenetic tree was constructed in MEGA v5.1 by the neighbour-joining method [33]. DNA polymorphism data, mean G+C content and phylogenetic calculations, including synonymous and non-synonymous substitutions, were performed using MEGA v5.1. A total of 1000 replicate bootstrap resampling analyses were performed to test the robustness of the nodes. Sequence similarities were corrected using the Jukes–Cantor correction. Comparison of inter-species similarity values for different genes was performed with a student’s t test using SPSS v13.0.

## Results

### Direct sequencing of *rrs* genes

Among the 138 clinical strains isolated from periodontal abscesses, 125 strains were identified by direct sequencing of *rrs* genes; the remaining 13 strains, had continuous overlapping signals in their chromatograms, which impaired the sequencing results and were solved by gene cloning. Bacterial genera and species distributions are shown in Table 2, with the dominant isolates belonging to *Prevotella* (89 isolates, 66%), *Veillonella* (19 isolates, 14%) and *Fusobacterium* (12 isolates, 8.9%).

**Table 2:** Characteristics of intragenomic heterogeneous 16S rRNA genes in clinical anaerobic strains.

Microorganisms	No. of strains	No. of strains with SNPs in 16S rRNA	No. of SNPs in 16S rRNA	No. strains with an indel in 16S rRNA
***Prevotella* genus**	**89**	**67**	**1–18**	**0–7**
*Prevotella intermedia*	29	22	1–12	0
*Prevotella nigrescens *	11	6	1–4	**7**
*Prevotella melaninogenica*	9	6	2–11	0
*Prevotella dentalis*	9	6	1–2	0
*Prevotella denticola*	9	8	4–12	0
*Prevotella buccae*	7	7	2–9	0
*Alloprevotella rava* ^**a**^	3	3	11–18	0
*Prevotella oris*	2	2	6–11	0
*Prevotella pallens*	2	2	6–7	0
*Candidatus Prevotella conceptionensis*	2	2	11–12	0
*Prevotella salivae*	1	0	-	0
*Prevotella fusca*	1	0	-	0
*Prevotella histicola*	1	1	2	0
*Prevotella oulorum*	1	1	7	0
*Prevotella veroralis*	1	0	-	0
*Prevotella multiformis*	1	1	8	0
***Veillonella* genus**	**19**	**19**	**2–14**	**0–6**
*Veillonella parvula/dispar*	15	15	3–14	6
*Veillonella sp*.	2	2	2–7	0
*Veillonella vegosae*	2	2	7–9	0
***Other genera***	**30**	**14**	**1–11**	**0**
*Fusobacterium nucleatum*	8	3	2–4	0
*Fusobacterium canifelinum*	4	3	2–3	0
*Bacteroides sp*.	3	2	2–8	0
*Actinomyces sp*.	3	0	-	0
*Campylobacter sp*.	2	0	-	0
*Dialister sp*.	2	1	1–2	0
*Peptostreptococcus sp*.	2	2	3	0
*Atopobium sp*.	1	0	-	0
*Bacillus sp*.	1	1	1	0
*Lachnoanaerobaculum sp*.	1	1	11	0
*Pseudoramibacter sp*.	1	0	-	0
*Shuttleworthia sp*.	1	0	-	0
*Selenomonas sp*.	1	1	2	0
**Total**	**138**	**100**		**13**

**^a^** *Alloprevotella rava* was previously classified as *Prevotella oral taxon 302*. Although it has been separated from the genus *Prevotella*, this strain was analysed together with the other *Prevotella spp*. in this study.

**Fig 1:**
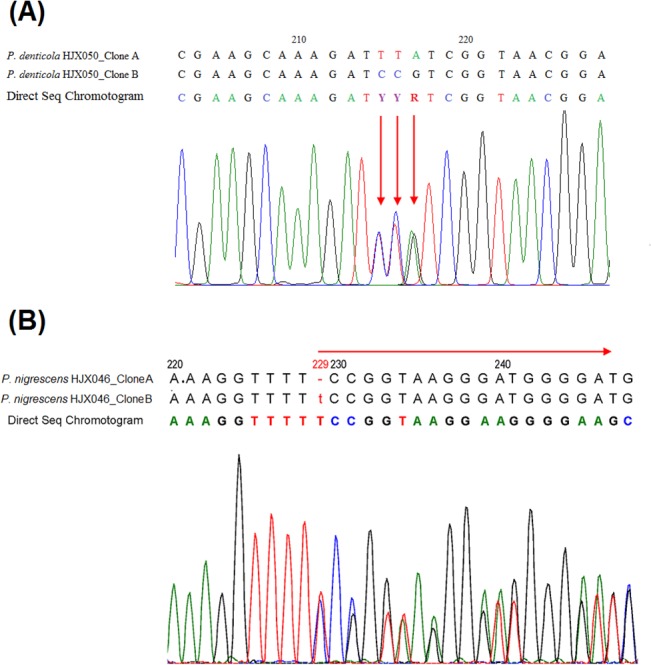
16S rRNA sequence trace data for the isolates *Prevotella denticola* HJX050 and *Prevotella nigrescens* HJX046, showing comparison sequence data for heterogeneous clones and direct sequencing with single ambiguous bases (A) or continuous ambiguous bases (B). *Prevotella nigrescens* HJX046 clone A had a deletion at nucleotide position 229.

When the direct sequencing chromatogram data were manually inspected, surprisingly, 100 anaerobic isolates (72%) had doublet sequencing signals in multiple locations, and 13 isolates (9.4%) had continuous ambiguous signals starting from certain regions, suggesting nucleotide polymorphisms (SNPs) or insertion/deletions (Indels), respectively, in the *rrs* genes. Two examples of overlapping ambiguous signals in chromatograms indicating SNPs (Fig 1A) and indels (Fig 1B) are shown. In the genera *Prevotella* and *Veillonella*, 75% (67/89) and 100% (19/19) of the clinical isolates, respectively, had SNPs in the *rrs* gene. The numbers of the SNPs ranged from 0 to 12 (0%-0.8% divergence) and from 2 to 14 (0.13%-0.93% divergence), respectively. In addition, heterogeneity was also observed among the isolates of genera *Fusobacterium*, *Bacteroides*, *Dialister*, *Peptostreptococcus*, *Bacillus*, *Lachnoanaerobaculum* and *Selenomonas* (Table 2). Seven *P*. *nigrescens* and 6 *Veillonella parvula/dispar* isolates had Indels in the *rrs* genes.

Amplification of *rrs* genes of 8 *Prevotella spp*. using Premix Ex-Taq enzyme and Q5 high-fidelity polymerase showed complete sequence match which implied that the doublet sequencing signals in direct sequencing were not enzyme related. In addition, the data showed that reference strain *P*. *melaninogenica* ATCC 25845 did not have any SNPs or Indels in the *rrs* genes, which was consistent with its four copies homogeneous *rrs* genes in the genome database (Genbank No. CP002122).

### *rrs* cloning and sequencing

Among the 13 isolates that were unidentified by direct sequencing, 6 were identified as *Veillonella parvula/dispar* and 7 were identified as *P*. *nigrescens* by *rrs* cloning. All 6 *Veillonella parvula/dispar* clinical isolates had the polymorphic allele “CG/AAA” at position 1137–1138 in the *rrs* gene (data not shown), and all 7 *P*. *nigrescens* isolates had a polymorphic T insertion at position 229 (according to the *E*. *coli rrs* numbering system) (Fig 1B). These intragenomic indels in *rrs* could explain the continuous doublet sequencing signals in chromatograms observed in the direct sequencing results.

To confirm the heterogeneity of the *rrs* genes as observed by direct PCR, we also performed *rrs* gene cloning for 2 *Prevotella buccae*, 2 *Prevotella denticola*, 8 *Prevotella intermedia*, 1 *Prevotella melaninogenica*, 1 *Prevotella oris*, 1 *Alloprevotella rava* and 7 *P*. *nigrescens* clinical isolates. By sequencing 8 to 14 clones of each isolates, more than one type of *rrs* was discovered. All intragenomic SNPs or indels revealed by cloning matched with the direct sequencing results. The data also showed that the clones of different *rrs* copies were unequal (Table 1). This was partially because some of the copies could be identical (see below). For example, in *P*. *intermedia* HJM057-2, *rrs-2* and *rrs-3* are identical (marked as copy A). Not surprisingly, the copy A was sequenced 4 out of 8 times when selection of clones, while other copies were sequenced fewer times (Table 1).

Furthermore, four copies of the *rrs*-ITS *genes* of 9 isolates of *P*. *intermedia* were separately amplified and sequenced. Unlike direct sequencing, which sequenced a mixture of all *rrs* copies, amplification of *rrs*-ITS *genes* directly measured each copy of *rrs*-ITS by flanking region primers and no doublet sequencing signals were found.

These clinical isolates contained 2 to 4 different types of *rrs* among 4 gene copies (Table 1), which were different from the identical *rrs* genes observed in the reference strains. The SNPs in the four copies of *rrs* were located at the same nucleotide positions observed by direct sequencing, further verifying the existence of intragenomic heterogeneity among different *rrs* sequences. An example of polymorphic nucleotide alignment for the 3 sequencing methods is shown in Fig 1. Totally 9 isolates of *P*. *intermedia* were sequenced by *rrs*-ITS separation sequencing (Table 1). Four *P*. *intermedia* isolates, HJX081-2, HJM050, HJM056 and HJM057-2, showed the same numbers of different types of *rrs* by both methods. For the isolate *P*. *intermedia* HJM069, *rrs-*ITS sequencing showed that it has 4 different copies of *rrs* genes, but only 3 different copies were revealed by selection of clones (Table 1). The discrepancy could be due to the limitation to only a few numbers of clones.

### Conserved genes of *Prevotella*

Partial sequences of five highly conserved protein-coding genes, *hsp60*, *recA*, *dnaJ*, *gyrB1* and *rpoB*, together with *rrs* were analysed and compared in 89 clinical isolates of *Prevotella*, 17 type strains of 14 *Prevotella* species and *Bacteroides fragilis* 638R as the out-group (S2 Table). Data from three *Alloprevotella rava* isolates formerly classified as *Prevotella sp*. *Taxon 302* were preserved in the study but excluded from analysis. The sequences used for alignments and phylogenetic tree construction were 546 bp, 600 bp, 777 bp, 1107 bp, 2028 bp and 1320 bp in length for *hsp60*, *recA*, *dnaJ*, *gyrB1*, *rpoB* and 16S rRNA, respectively.

For all genes, the intra-species comparisons demonstrated high similarities among species where more than one strain was available, except for *P*. *melaninogenica*. Percentage sequence similarities based on pairwise comparisons for all six genes were analysed (S3 Table). The ranges of intra-species nucleotide similarities for *hsp60*, *recA*, *dnaJ*, *gyrB1*, *rpoB* and *rrs* were 94.3–100% (mean±SEM: 98.6±1.1%), 90.8–100% (98.1±1.5%), 94.9–100% (98.3±0.97%), 95.3–100% (98.5±1.1%), 93.5–100% (98.5±1.5%) and 98.6–100% (99.5±0.31%), respectively, if *P*. *melaninogenica* was divided and analysed as two subspecies as described below. The ranges of inter-species similarities for *hsp60*, *recA*, *dnaJ*, *gyrB1*, *rpoB* and *rrs* were 68.7–91.6% (mean±SEM: 79.3±4.2%), 67.7–95.3% (77.0±5.5%), 59.7–97.9% (75.5±5.6%), 69.6–90.3% (77.6±4.3%), 70.1–89.6% (79.0±4.3%), and 85.5–98.3% (91.4±2.7%) (Table 3), respectively. All inter-species similarity values for *hsp60*, *recA*, *dnaJ*, *gyrB1* and *rpoB* were significantly lower than for *rrs* (P<0.001), and parsimony-informative sites were higher than for *rrs* (23.9%), resulting in greater discriminatory power in species identification compared with 16S rRNA. The average dN/dS values are shown in Table 3. All values are less than 0.2, indicating that these genes are under purifying selection pressure.

**Table 3:** Analysis of 106 *hsp60*, *recA*, *dnaJ*, *gyrB1*, *rpoB* and 16S rRNA gene sequences from *Prevotella*.

Sequence information	*hsp60*	*recA*	*dnaJ*	*gyrB1*	*rpoB*	16S rRNA
No. of nucleotide sites	546	600	777	1107	2028	1320
Mean G+C content (mol%)	47.10%	51.30%	52.70%	50.50%	46.70%	55.50%
No. of polymorphic sites	246	310	481	511	1017	323
No. of parsimony-informative sites	241 (44.1%)	299 (49.8%)	465 (59.8%)	506 (45.7%)	918 (45.3%)	315 (23.9%)
No. of nucleotide differences:					
Range	0–171	0–194	0–314	0–337	0–650	0–183
Mean±SEM	97.0±5.3	115.9±6.6	162.4±5.8	221.0±6.5	391.3±10.0	110.1±6.4
Intra-species±SD	7.6±6.0	11.3±9.2	13.1±7.57	16.6±11.7	30.7±30.8	6.6±4.1
Inter-species±SD	113.0±22.7	138.0±33.1	190.4±43.5	248.5±47.9	425.1±87.0	113.0±36.1
Jukes–Cantor distance (d):					
Overall mean±SEM	0.209±0.014	0.232±0.016	0.389±0.023	0.239±0.008	0.229±0.007	0.091±0.006
Transition:transversion ratio (R)	1.8	1.85	1.26	1.65	1.82	1.49
dS*	1.225±0.064	0.990±0.116	0.782±0.113	1.103±0.117	1.431±0.052	NA
dN*	0.052±0.009	0.056±0.008	0.127±0.011	0.055±0.007	0.063±0.005	NA
dN/dS	0.042	0.057	0.162	0.05	0.044	NA

*Synonymous substitutions per site (dS) and non-synonymous substitutions per site (dN) (means±SEM) were determined by the Nei–Gojobori method using the Jukes–Cantor distance. NA, not applicable.

Although *recA* and *dnaJ* were the most informative genes (having the highest parsimony-informative sites), they had difficulty in discriminating several pairs of species. The *dnaJ* sequence similarities for the *P*. *intermedia*/*P*. *nigrescens* and the *P*. *multiformis*/*P*. *denticola* pairs were uncommonly high and even higher than some intra-species similarity levels for these species (S3 Table, dnaJ sheet). Similar results were observed in the *P*. *intermedia*/*P*. *nigrescens* and *P*. *fusca*/*P*. *melaninogenica* pairs for *recA* (S3 Table, recA sheet) and in the *P*. *multiformis/P*. *denticola* pair for *hsp60* (S3 Table, hsp60 sheet). Nucleotide sequence similarities for *gyrB1* and *rpoB* in particular had sufficient differences between the intra- and inter-species similarities for the accurate classification of *Prevotella* species, making them suitable as classification markers (S3 Table). We propose that thresholds of 93% and 91% for *gyrB1* and *rpoB*, respectively, could be used for species classification in the *Prevotella* genus. The thresholds were set as the average similarity values of the maximum inter-species and the minimum intra-species.

**Fig 2:**
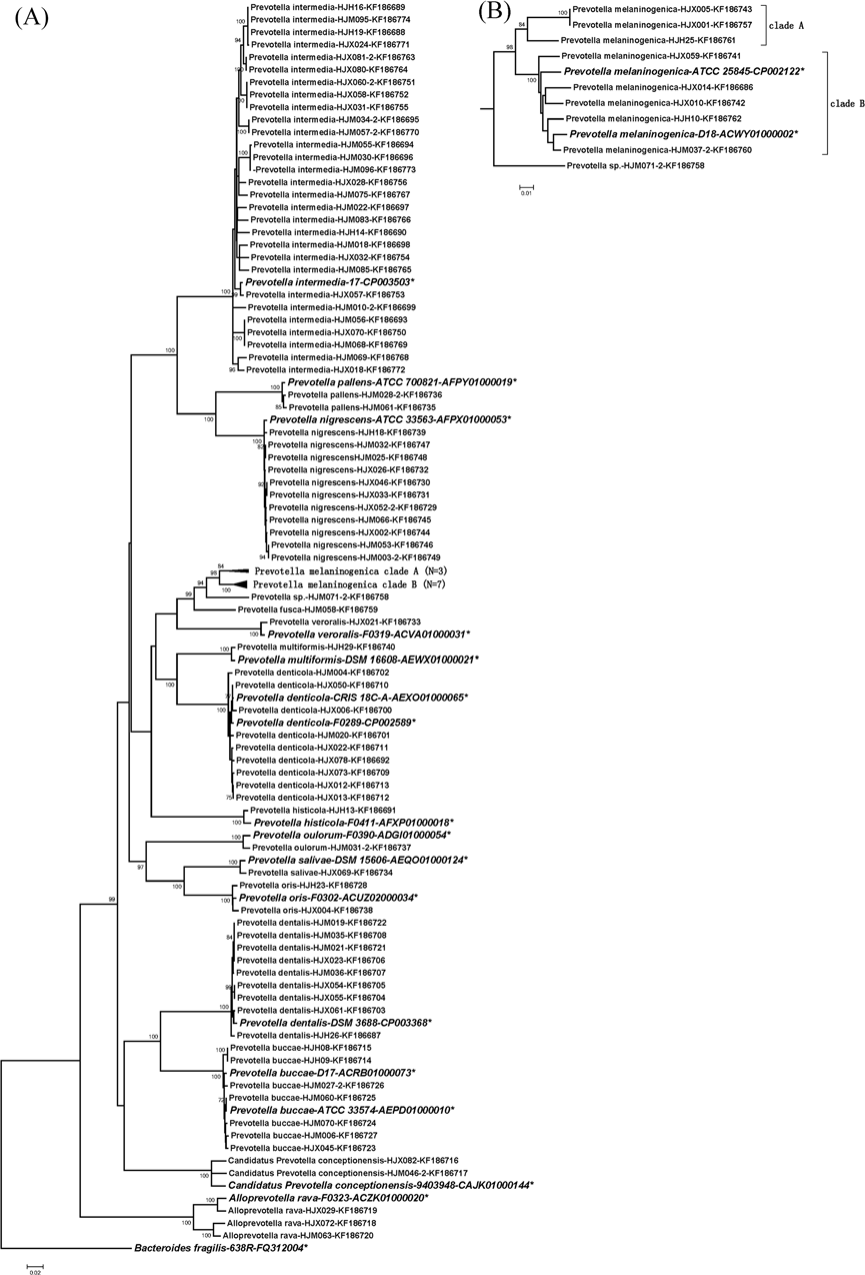
Phylogenetic trees based on the concatenated sequences of the *gyrB1* gene. Detailed phylogenetic trees of *P*. *melaninogenica* strains are shown in Figure (B). The trees were constructed by the neighbour-joining (NJ) method. The numbers at nodes indicate the percentage bootstrap values of 1000 replicates (>70%). Bars indicate the expected nucleotide substitutions per site. * represents reference strains.

**Fig 3:**
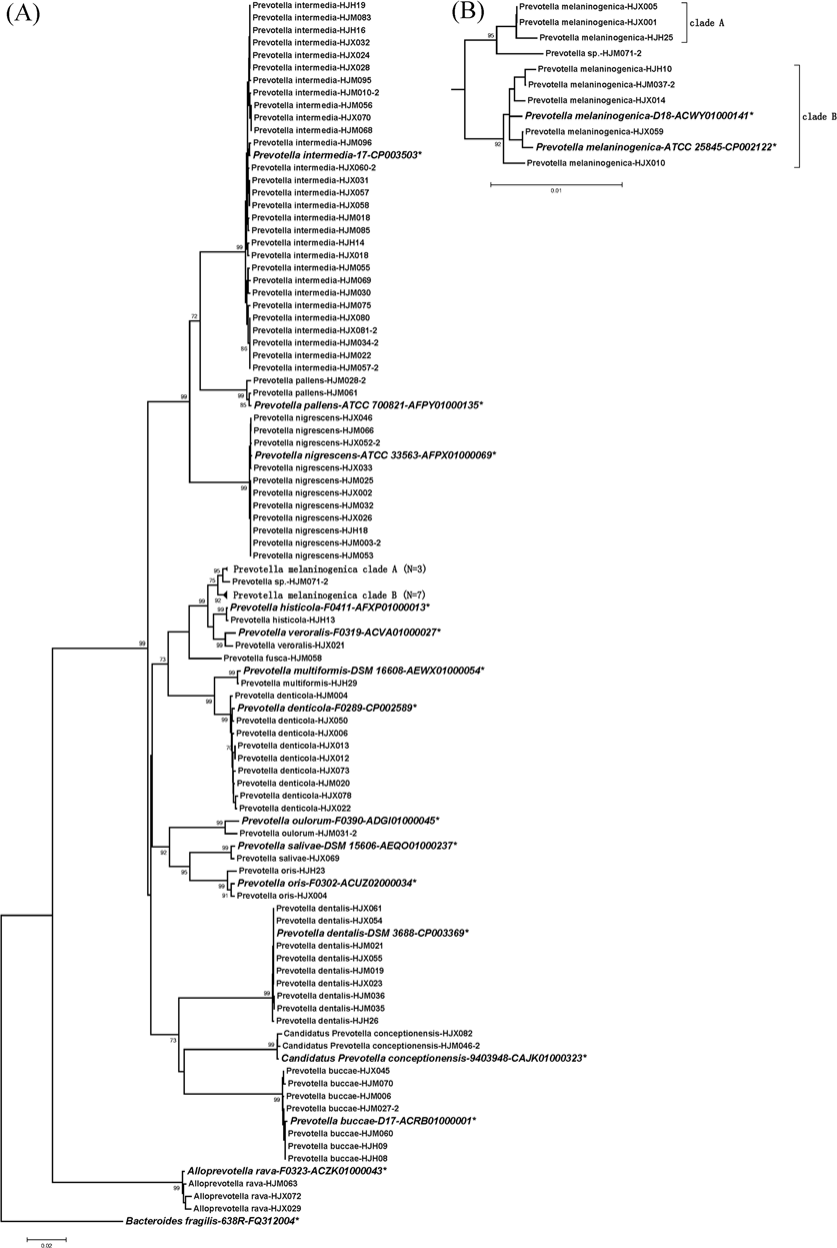
Phylogenetic trees based on the concatenated sequences of the 16S rRNA gene. Detailed phylogenetic trees of *P*. *melaninogenica* strains are shown in Figure (B). The trees were constructed by the neighbour-joining (NJ) method. The numbers at nodes indicate the percentage bootstrap values of 1000 replicates (>70%). Bars indicate the expected nucleotide substitutions per site. * represents reference strains.

**Fig 4:**
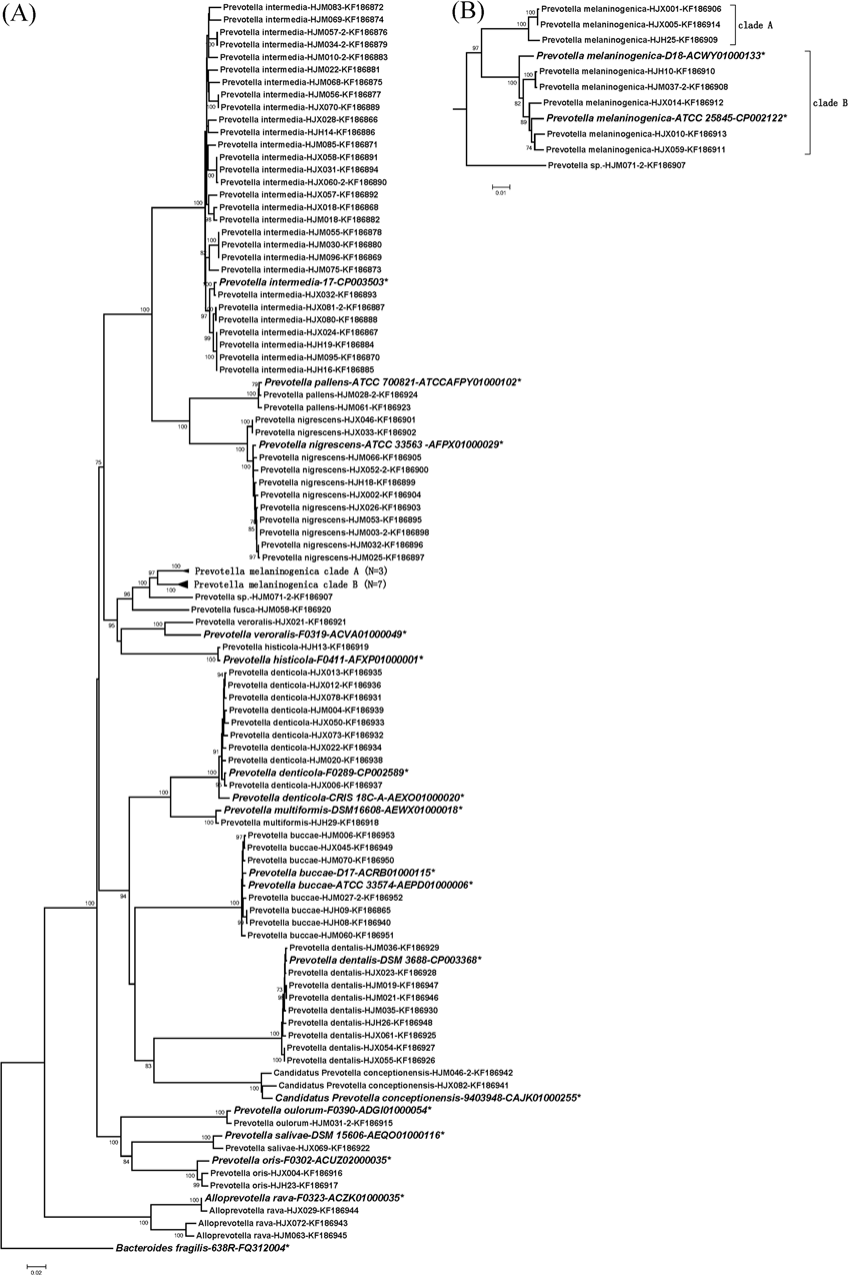
Phylogenetic trees based on the concatenated sequences of the *rpoB* gene. Detailed phylogenetic trees of *P*. *melaninogenica* strains are shown in Figure (B). The trees were constructed by the neighbour-joining (NJ) method. The numbers at nodes indicate the percentage bootstrap values of 1000 replicates (>70%). Bars indicate the expected nucleotide substitutions per site. * represents reference strains.

In agreement with the alignment analysis, the phylogenetic trees constructed for each gene were very stable at the intra-species level. Isolates from the same species, except *P*. *melaninogenica*, clustered tightly together with very high bootstrap values (>95%) (Figs 2, 3, 4 and S1, S2, S3 Figs). Nevertheless, 9 clinical *P*. *melaninogenica* isolates and 2 type strains (ATCC25845 and D18) clearly clustered into 3 branches, with the first branch including *P*. *melaninogenica* HJM071-2 only, a second branch having three isolates and the third branch for the other 5 clinical isolates and the 2 type strains. The sequence similarities between these branches of *hsp60*, *recA*, *dnaJ*, *gyrB1* and *rpoB* were 89–94.0%, 87.5–95.8%, 84.1–88.9%, 89.3–94.5% and 91.3–94.2%, respectively (data not shown), which are between the inter- and intra-species similarities of *Prevotella*. Because the phylogenetic relationships are stable (bootstrap >80%) in all trees except *recA*, we reclassified these isolates into 2 sub-species, specifically clades A and B, and separated strain *P*. *melaninogenica* HJM071-2 alone (Figs 2, 3, 4 and S1, S2 Figs).

At the genus level, the phylogenetic trees constructed for different genes gave similar results. *P*. *intermedia*, *P*. *nigrescens* and *P*. *pallens* were consistently clustered in one group for all genes (all bootstrap values >94%). *P*. *oris*, *P*. *salivae* and *P*. *oulorum* had smaller genetic distances and thus tended to cluster together according to the *gyrB1* (Fig 2), 16S rRNA (Fig 3), *rpoB* (Fig 4) and *hsp60* (S1 Fig) genes. *P*. *melaninogenica*, *P*. *fusca*, *P*. *veroralis* and *P*. *histicola* were closely related in the *hsp60* (S1 Fig), 16S rRNA (Fig 3) and *rpoB* (Fig 4) trees. There was a small difference between genes regarding the phylogenetic position of *P*. *fusca*, as this strain was close to *P*. *melaninogenica* in all gene trees except the 16S rRNA tree (Fig 3). Another group consisted of *P*. *buccae*, *P*. *dentalis* and *Candidatus Prevotella conceptionensis*, but this group was rather unstable. *P*. *multiformis* and *P*. *denticola* were closely clustered in all trees except for the *recA* tree (S3 Fig). The grouping results could explain the uncommonly high sequence similarity values for some of the aforementioned genes for the *P*. *intermedia*/*P*. *nigrescens*, *P*. *fusca*/*P*. *melaninogenica* and *P*. *multiformis*/*P*. *denticola* pairs (S3 Table, *recA* and *dnaJ* sheets), suggesting that gene recombination was more likely to have occurred in closely grouped taxa. The phylogenetic analysis also indicated a higher evolutionary rate for the five housekeeping gene sequences compared to the *rrs* gene sequence.

## Discussion

The *Prevotella* genus was systematically reclassified from *Bacteroides* in 1990 by Shah and Collins [34]. With increasing discoveries of novel *Prevotella* species and in-depth studies of intestinal and oral flora, accurate classification and identification of these causative strictly anaerobic pathogens has gradually received greater attention [35, 36]. Currently, clinical identification of anaerobic bacteria mainly relies on conventional biochemical phenotyping [37], *rrs* sequencing [21, 23] or bacterial fingerprint identification by matrix-assisted laser desorption ionisation time-of-flight mass spectrometry (MALDI-TOF MS) [38–41]. Sequence analysis of *rrs* remains inevitable in *rrs-*based methods, gold or reference methods and comparison studies as well as for difficult-to-identify isolates.

In our study, many clinical opportunistic anaerobic pathogens were shown to have intra-species *rrs* heterogeneity, contrasting with the 4 homogeneous *rrs* genes in the genomes of the reference strains. With the exception of *Veillonella* [19, 25], this is the first report of this variability. We believe the discrepancy between the reference strains may comprise two aspects. The data showed that 22 of 29 *P*. *intermedia* had SNPs in their 16S rRNA genes (Table 2). In other words, there are still a certain number of isolates whose *rrs* genes are homogeneous. Second, differences in geography, site of infection and antibiotic usage could contribute to the discrepancy. The percentage and the type (Indel or SNP) of heterozygosity of *rrs* genes will directly affect the accuracy and feasibility of the classification and identification of strains. In this study, the intra-genomic 16S rRNA sequence similarity of most heterozygous *Prevotella* strains, such as *P*. *denticola*, *P*. *conceptionensis and P*. *intermedia*, was only 99.1%. For *Alloprevotella rava* stains, the similarity was even lower. In diagnostic practice, continuous doublet signals caused by indels, noted in our study in 7 *P*. *nigrescens* and 6 *Veillonella parvula/dispar* isolates, are a challenge not only in terms of the feasibility of direct PCR-sequencing methods and automated clinical instruments but also to the researcher who might repeatedly suspect contamination of the strains. If indels appear to be stable at certain positions, a compromise could be to use other shorter viable regions of *rrs* and avoid heterogeneous regions. The identification accuracy of other commonly utilised microbiological methods, such as PCR-TTGE and PCR-RFLP, would also be affected by heterozygosity, with results consisting of multiple erroneous bands. For microbial community methods, such as PGGE and 16S rDNA high-throughput sequencing, the heterogeneity of *rrs* would result in overestimation of the diversity of the population, particularly for those strains with higher intra-species diversity than inter-species diversity [42]. Heterogeneity of *rrs* genes appears to be correlated with the complexity of the microbiota environment and may contribute to survival under stress [43]. Periodontal abscesses in clinical patients usually have multiple bacterial infections and are under stress from antibiotics or host immune responses. It appears rational to propose that clinical strains isolated from microflora infections such as periodontitis are more likely evolve or mutate their *rrs* genes to adapt to the environment [25, 44–46]. This study performed *rrs* cloning to confirm the heterogeneity of the *rrs* gene. The method could not determine accurate copy numbers, whether there were 4 copies of *rrs*, such as in *Prevotella intermedia*, or greater or fewer copies. Selection of clones satisfies the binomial distribution; therefore, limitation to only a few clones cannot predict the distribution of different copies in the isolates. Genomic sequencing and analysis is needed to study the exact numbers and the distribution of *rrs* genes.

In this study, in *Prevotella*, 75% of 89 isolates exhibited intra-genomic heterogeneity, which included multiple species. Isolates of the genera *Streptomyces* and *Aeromonas* were previously shown to possess only 21% and 6.9% intra-genomic heterogeneity for *rrs*, respectively, based on sequencing patterns and RFLP analysis [43, 47]. Although different methods were used, *Prevotella* and the clinically relevant anaerobes examined here demonstrated the highest occurrences of intra-genomic heterogeneity compared with these previously described genera.

Previously Sakamoto et al. analysed *hsp60* sequences in 48 *Prevotella* strains from 38 species [48] and concluded that *hsp60* could be an alternative phylogenetic marker in *Prevotella*. Our study analysed the *recA*, *dnaJ*, *gyrB1*, *hsp60* and *rpoB* genes in a greater number of clinical isolates and type strain sequences, which permitted better evaluation and comparison of classification markers. Although *hsp60* sequences could accurately classify most species of *Prevotella*, the high similarity of *P*. *multiformis* and *P*. *denticola* isolates impeded its accuracy in classification. Instead, *gyrB1* and *rpoB* showed to be better markers for classification in *Prevotella* based on intra- and inter-species comparisons. However, based on phylogenetic tree *rpoB* proved to be reliable phylogenetic marker for the genus *Prevotella*.

We recognise several limitations to this study. All clinical strains were isolated from one clinical centre, and some species, such as *P*. *fusca*, comprised only a limited number of clinical isolates. Although we added certain type strains that were available from databases to the study, some findings and conclusions must be verified using additional clinical isolates and strains from other locations. Although we verified the homogeneous *rrs* genes of the only available reference strain, *P*. *melaninogenica* ATCC 25845, we could not test the others reference strains due to the limitation of resources. The homogeneous *rrs* genes of the others reference strains are needed to be further verified. Furthermore, because the nucleotide database for housekeeping genes such as *gyrB1*, *rpoB* and *hsp60* is quite small, particularly for newly classified *Prevotella* species, identification and classification using these genes should be supported by a larger database. Hopefully, when the whole genome of a bacteria is sequenced, all housekeeping genes can be acquired simultaneously. With the expansion of the Human Microbiome Project and the advent of bacterial whole genomic sequencing, inadequate database problems will gradually be diminished. For instance, in this study, we did not amplify or sequence the housekeeping genes of any reference strains ourselves; instead, these genes were analysed and extracted from the genome database.

## Conclusions

In conclusion, 16S rRNA gene heterogeneity was apparent in clinical, strictly anaerobic oral bacteria, particularly in the genera *Prevotella* and *Veillonella*, which could interfere with classification and identification methods based on 16S rRNA genes. The housekeeping gene sequences *rpoB* and *gyrB1* were demonstrated to be alternative classification markers to the species level based on intra- and inter-species comparisons, whereas based on phylogenetic tree *rpoB* proved to be reliable phylogenetic marker for the genus *Prevotella*. We propose nucleotide similarity thresholds for species classification for *rpoB* and *gyrB1* as 91%, and 93%, respectively.

## Supporting Information

S1 Fig Phylogenetic trees based on the concatenated sequences of the *hsp60* gene. The trees were constructed by the neighbour-joining (NJ) method. The numbers at nodes indicate the percentage bootstrap values of 1000 replicates (>70%). Bars indicate the expected nucleotide substitutions per site. * represents reference strains.
(TIF)

S2 Fig Phylogenetic trees based on the concatenated sequences of the *dnaJ* gene. The trees were constructed by the neighbour-joining (NJ) method. The numbers at nodes indicate the percentage bootstrap values of 1000 replicates (>70%). Bars indicate the expected nucleotide substitutions per site. * represents reference strains.
(TIF)

S3 Fig Phylogenetic trees based on the concatenated sequences of the *recA* gene. The trees were constructed by the neighbour-joining (NJ) method. The numbers at nodes indicate the percentage bootstrap values of 1000 replicates (>70%). Bars indicate the expected nucleotide substitutions per site. * represents reference strains.
(TIF)

S1 Table Primers and PCR conditions used in the study. (DOCX)

S2 Table Bacterial strains used in this study and their accession numbers in the databases. (DOCX)

S3 Table Sequence similarity based on pairwise comparisons of the 16S rRNA, *gyrB1, hsp60, rpoB, recA* and *dnaJ* sequences of *Prevotella* species. Green, pink, blue, yellow and open-shaded rectangles indicate >50%, >60%, >70%, >80% and >90% sequence similarity, respectively. *** indicates that data are not available because there is only one strain in the genus.
(XLSX)
