# Supplementary figures and images for: Intra-Genomic Heterogeneity in 16S rRNA Genes in Strictly Anaerobic Clinical Isolates from Periodontal Abscesses
Source: PLoS One. 2015 Jun 23;10(6):e0130265. doi: 10.1371/journal.pone.0130265 (PMC4477887; doi:10.1371/journal.pone.0130265)

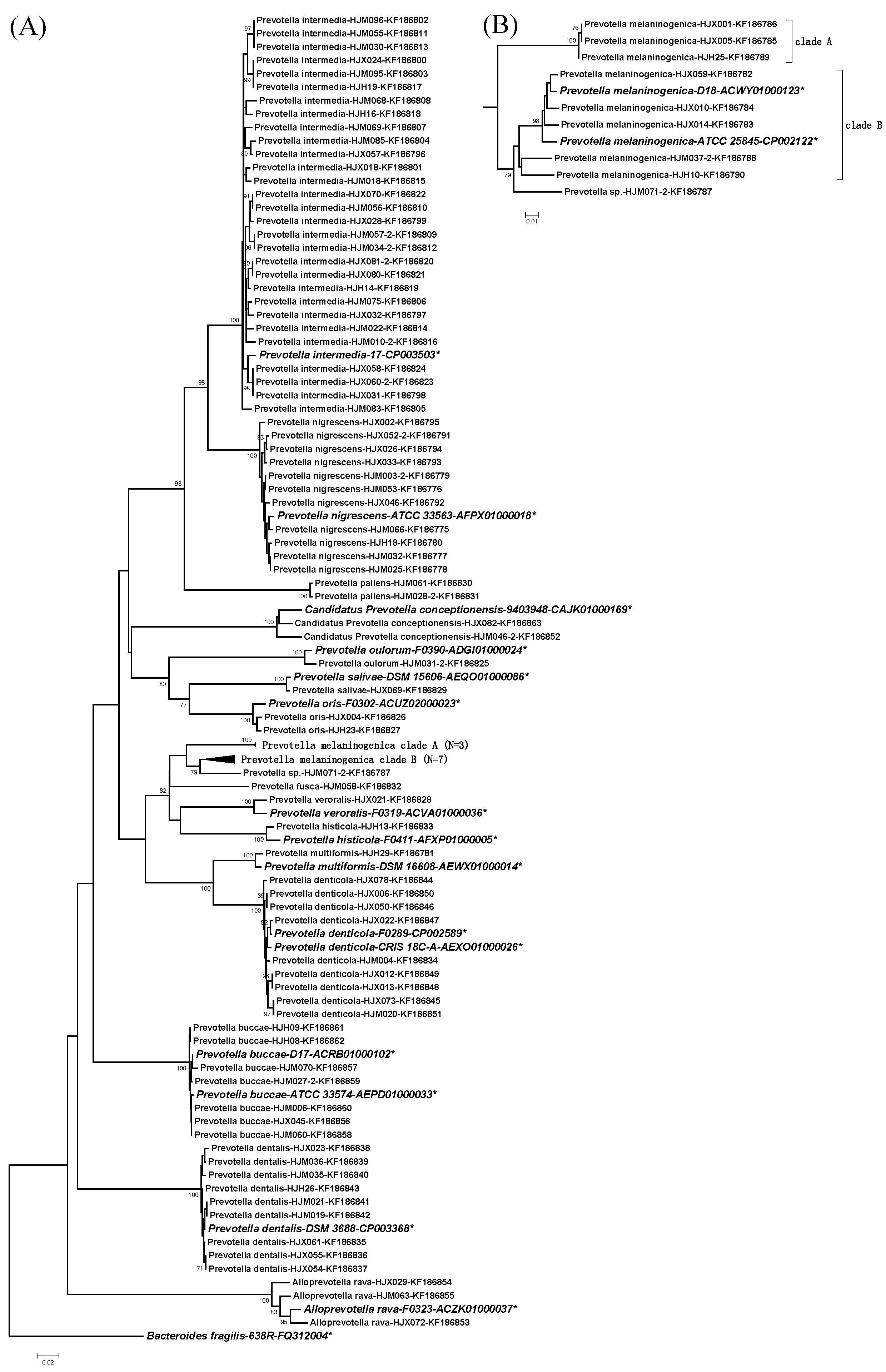

Supplement: S1 Fig — The trees were constructed by the neighbour-joining (NJ) method. The numbers at nodes indicate the percentage bootstrap values of 1000 replicates (>70%). Bars indicate the expected nucleotide substitutions per site. * represents reference strains. (TIF) [file pone.0130265.s001.tif]

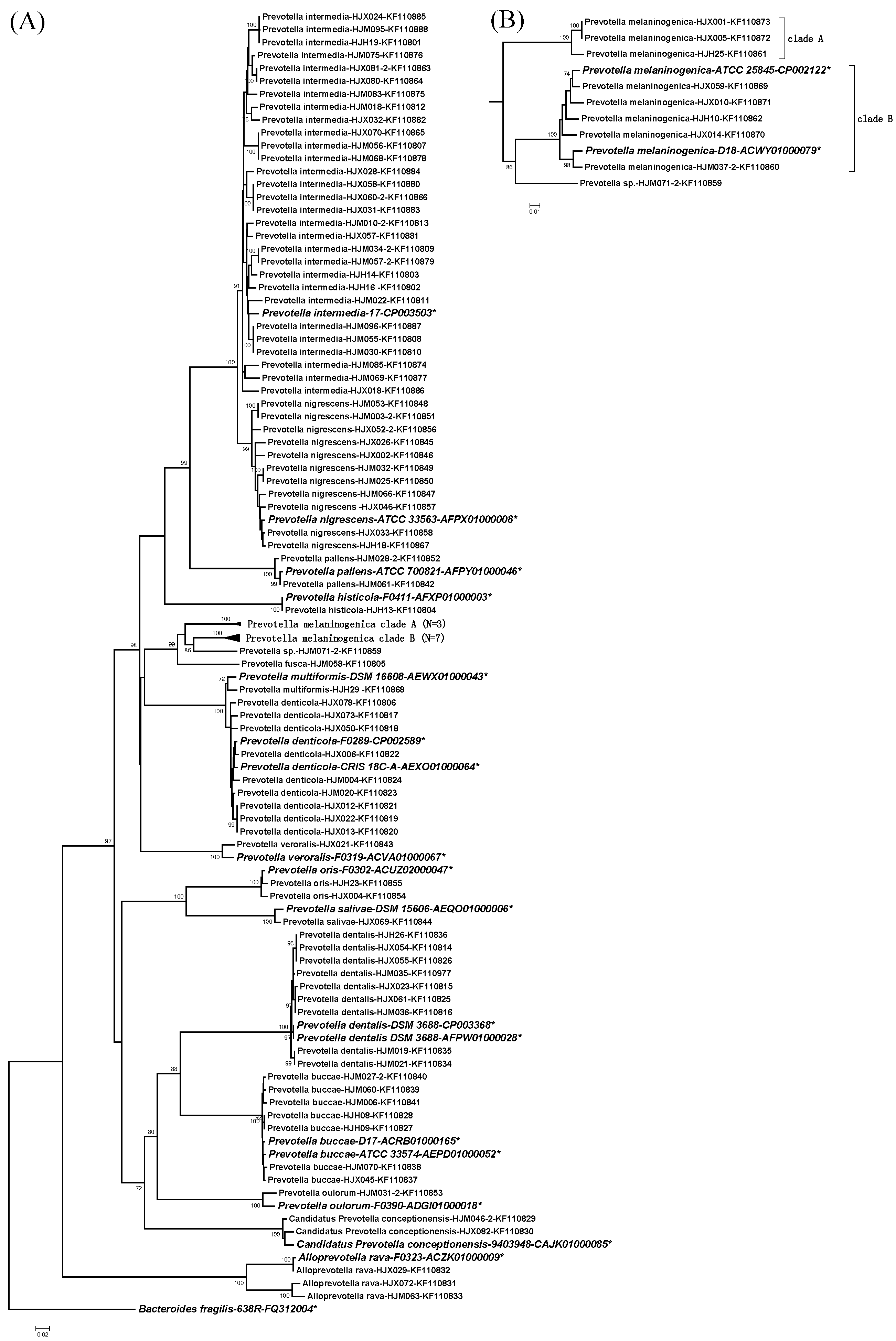

Supplement: S2 Fig — The trees were constructed by the neighbour-joining (NJ) method. The numbers at nodes indicate the percentage bootstrap values of 1000 replicates (>70%). Bars indicate the expected nucleotide substitutions per site. * represents reference strains. (TIF) [file pone.0130265.s002.tif]

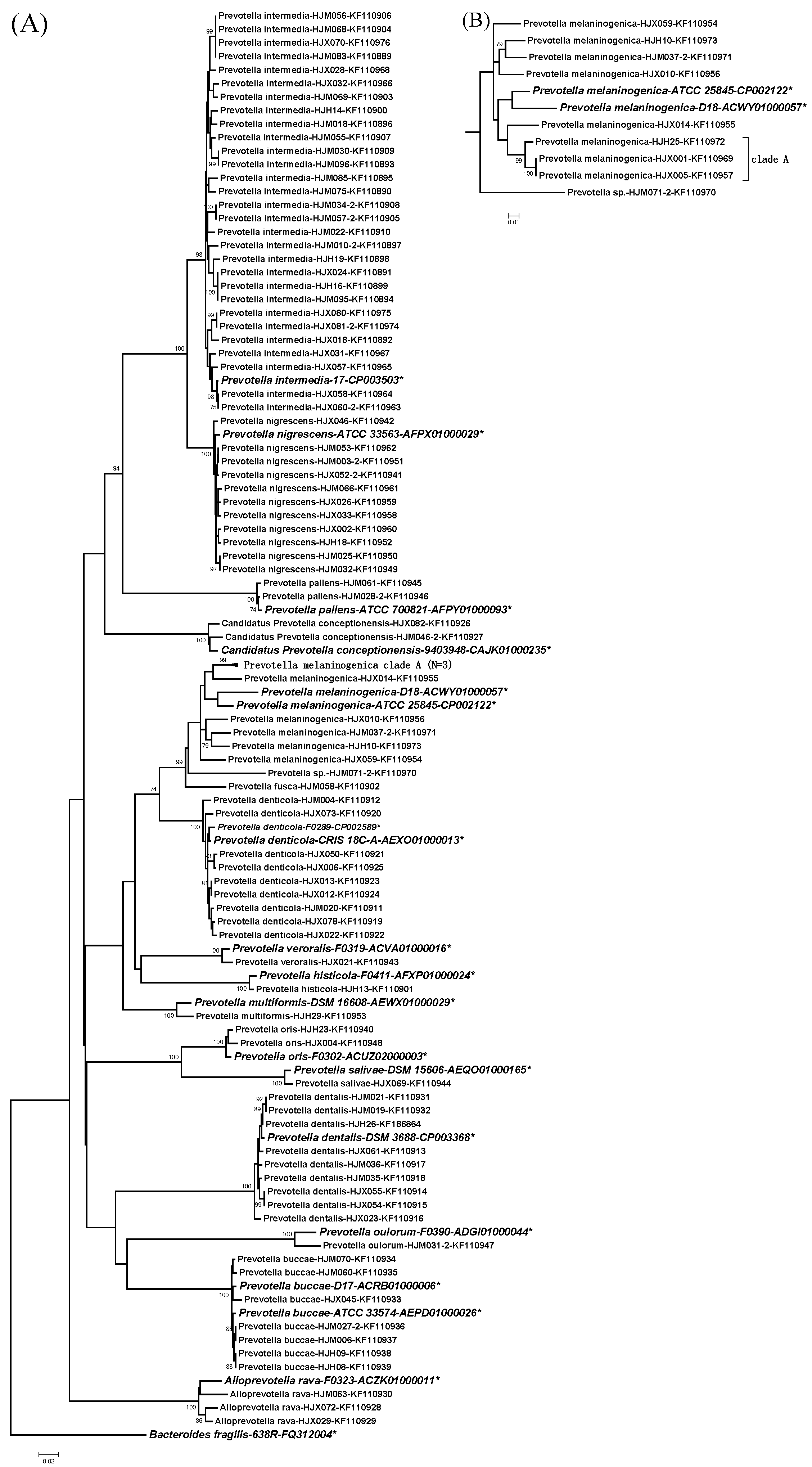

Supplement: S3 Fig — The trees were constructed by the neighbour-joining (NJ) method. The numbers at nodes indicate the percentage bootstrap values of 1000 replicates (>70%). Bars indicate the expected nucleotide substitutions per site. * represents reference strains. (TIF) [file pone.0130265.s003.tif]
