# Supplementary material for: Intra-Genomic Heterogeneity in 16S rRNA Genes in Strictly Anaerobic Clinical Isolates from Periodontal Abscesses
Source: PLoS One. 2015 Jun 23;10(6):e0130265. doi: 10.1371/journal.pone.0130265 (PMC4477887; doi:10.1371/journal.pone.0130265)
Supplement: S1 Table — (DOCX) [file pone.0130265.s004.docx]

**Supplementary Table 1.** Primers and PCR conditions used in the study

| Primers | Sequences (5'-3') | PCR condition | Amplification length | Resources |
| --- | --- | --- | --- | --- |
| *rpoB*-1F  (Forward) | CARTTYATGGAYCARACIAAYCC | 35cycles：94˚Cx30s, 50˚Cx30sec, 72˚Cx2min | *rpoB*  2028bp | This study |
| *rpoB*-3623R  (Reverse) | DGCCCANACYTCCATYTCWCC |  |  |  |
| H729  (Forward) | **CGCCAGGGTTTTCCCAGTCACGAC**GAI III GCI GGI GAY GGI ACI ACI AC | 35cycles：94˚Cx30s, 50˚Cx30sec, 72˚Cx1min | *hsp60*  546bp | Brousseau et al**†** |
| H730  (Reverse) | **AGCGGATAACAATTTCACACAGGA**YKI YKI TCI CCR AAI CCI GGI GCY TT |  |  |  |
| *recA*-1F  (Forward) | **GCAGCGATGTCAAAAATAGAAA**RGAYTTYGGNAARGGNKCNATYATG | 35cycles：94˚Cx30s, 58˚Cx30sec, 72˚Cx1min | *recA*  600bp | This study |
| *recA*-1R  (Reverse) | **CGAAGAGAATGTCGAATTCTACCTTA**CGGAANGGNGGNGCNACYTTRTT |  |  |  |
| *dnaJ* -1F  (Forward) | **AGAAGGGCTATCGCAAACTA**GCYATHAARTWYCAYCCIGA | 35cycles：94˚Cx30s, 50˚Cx30sec, 72˚Cx1min | *dnaJ*  747bp | This study |
| *dnaJ* -1R  (Reverse) | **TACCACGCAGGCGGAGCGT**YKTNCCIGGYTGIGTICC |  |  |  |
| *gyrB*-1F  (Forward) | **GAAGTCATCATGACCGTTCT**NCAYGCNGGNGGNAARTTYG | 35cycles：94˚Cx30s, 50˚Cx30sec, 72˚Cx1.5min | *gyrB*  1098bp | This study |
| *gyrB*-2R  (Reverse) | **AGCAGGGTACGGATGTGCGAG**CCRTCNACRTCNGCRTCNGTCAT |  |  | Yamamoto& Harayama **‡** |
| Inter_rrn1-F(Forward) | GTGCCCTGTGTCCTCTTTCA | 35cycles：94˚Cx30s, 55˚Cx30sec, 72˚Cx2min | *rrn1* | This study |
| Inter_rrn2-F(Forward) | ACACCCTATGACAGAAGCACACT |  | *rrn2* |  |
| Inter_rrn3-F(Forward) | TGTCCAAAATCTGATGCGATAG |  | *rrn3* |  |
| Inter_rrn4- F(Forward) | TGCCGAACAAAACACACCTA |  | *rrn4* |  |
| 23S_1-60-R(Reverse) | GGCTTATCGCAGCTTATCACG |  | *rrn1-4* |  |
| *rpoB-s*eq2100F | TTCCGNMGHACNAAYCARAAYATG |  | *rpoB* sequencing |  |
| *hsp60*-seq1F | CGCCAGGGTTTTCCCAGTCACGAC |  | *hsp60* sequencing |  |
| *hsp60*-seq1R | AGCGGATAACAATTTCACACAGGA |  |  |  |
| *recA*-seq1F | GCAGCGATGTCAAAAATAGAAA |  | *recA* sequencing |  |
| *recA*-seq1R | CGAAGAGAATGTCGAATTCTACCTTA |  |  |  |
| *dnaJ* -seq1F | AGAAGGGCTATCGCAAACTA |  | *dnaJ* sequencing |  |
| *dnaJ* -seq1R | TACCACGCAGGCGGAGCGT |  |  |  |
| *gyrB*-seq1F | GAAGTCATCATGACCGTTCT |  | *gyrB* sequencing |  |
| *gyrB*-seq2R | AGCAGGGTACGGATGTGCGAG |  |  |  |

† Ronald Brousseau, Janet E. Hill, Gabrielle Préfontaine, Swee-Han Goh, Josée Harel, Sean M. Hemmingsen. (2001) Streptococcus suis Serotypes Characterized by Analysis of Chaperonin 60 Gene Sequences. *Appl Environ Microbiol*.

‡ Sakamoto M, Ohkuma M. (2011) Identification and classification of the genus Bacteroides by multilocus sequence analysis. *Microbiology*.
