# Supplementary material for: Intra-Genomic Heterogeneity in 16S rRNA Genes in Strictly Anaerobic Clinical Isolates from Periodontal Abscesses
Source: PLoS One. 2015 Jun 23;10(6):e0130265. doi: 10.1371/journal.pone.0130265 (PMC4477887; doi:10.1371/journal.pone.0130265)
Supplement: S2 Table — (DOCX) [file pone.0130265.s005.docx]

**Supplementary Table 2. Bacterial strains used in this study and their accession numbers in the databases**

| **Reference strains** | ***rpoB* gene Accession number** | ***gyrB1* gene Accession number** | ***recA* gene Accession number** | ***hsp60* gene Accession number** | ***dnaJ* gene Accession number** |
| --- | --- | --- | --- | --- | --- |
| *Prevotella intermedia* 17 | CP003503 | CP003503 | CP003503 | CP003503 | CP003503 |
| *Prevotella melaninogenica* ATCC 25845 | CP002122 | CP002122 | CP002122 | CP002122 | CP002122 |
| *Prevotella melaninogenica* D18 | ACWY01000133 | ACWY01000002 | ACWY01000057 | ACWY01000123 | ACWY01000079 |
| *Prevotella denticola* F0289 | CP002589 | CP002589 | CP002589 | CP002589 | CP002589 |
| *Prevotella denticola* CRIS 18C-A | AEXO01000020 | AEXO01000065 | AEXO01000013 | AEXO01000026 | AEXO01000064 |
| *Prevotella dentalis* DSM 3688 | CP003368 | CP003368 | CP003368 | CP003368 | CP003368 |
| *Prevotella nigrescens* ATCC 33563 | AFPX01000029 | AFPX01000053 | AFPX01000029 | AFPX01000018 | AFPX01000008 |
| *Prevotella oris* F0302 | ACUZ02000035 | ACUZ02000034 | ACUZ02000003 | ACUZ02000023 | ACUZ02000047 |
| *Prevotella buccae* ATCC 33574 | AEPD01000006 | AEPD01000010 | AEPD01000026 | AEPD01000033 | AEPD01000052 |
| *Prevotella buccae* D17 | ACRB01000115 | ACRB01000073 | ACRB01000006 | ACRB01000102 | ACRB01000165 |
| *Prevotella histicola* F0411 | AFXP01000001 | AFXP01000018 | AFXP01000024 | AFXP01000005 | AFXP01000003 |
| *Prevotella oulorum* F0390 | ADGI01000054 | ADGI01000054 | ADGI01000044 | ADGI01000024 | ADGI01000018 |
| *Prevotella pallens* ATCC 700821 | AFPY01000102 | AFPY01000019 | AFPY01000093 | AFPY01000081 | AFPY01000046 |
| *Prevotella salivae* DSM 15606 | AEQO01000116 | AEQO01000124 | AEQO01000165 | AEQO01000086 | AEQO01000006 |
| *Prevotella multiformis* DSM 16608 | AEWX01000018 | AEWX01000021 | AEWX01000029 | AEWX01000014 | AEWX01000043 |
| *Prevotella veroralis* F0319 | ACVA01000049 | ACVA01000031 | ACVA01000016 | ACVA01000036 | ACVA01000067 |
| *Alloprevotella rava* F0323 | ACZK01000035 | ACZK01000020 | ACZK01000011 | ACZK01000037 | ACZK01000009 |
| *Candidatus Prevotella conceptionensis* 9403948 | CAJK01000255 | CAJK01000144 | CAJK01000235 | CAJK01000169 | CAJK01000085 |
| *Bacteroides fragilis* 638R | FQ312004 | FQ312004 | FQ312004 | FQ312004 | FQ312004 |
| **Clinical strains** | ***rpoB* gene Accession number** | ***gyrB1* gene Accession number** | ***recA* gene Accession number** | ***hsp60* gene Accession number** | ***dnaJ* gene Accession number** |
| *Alloprevotella rava* HJX072 | KF186943 | KF186718 | KF110928 | KF186853 | KF110831 |
| *Alloprevotella rava* HJM063 | KF186945 | KF186720 | KF110930 | KF186855 | KF110833 |
| *Alloprevotella rava* HJX029 | KF186944 | KF186719 | KF110929 | KF186854 | KF110832 |
| *Candidatus Prevotella conceptionensis* HJM046 | KF186942 | KF186717 | KF110927 | KF186852 | KF110829 |
| *Candidatus Prevotella conceptionensis* HJX082 | KF186941 | KF186716 | KF110926 | KF186863 | KF110830 |
| *Prevotella buccae* HJH08 | KF186940 | KF186715 | KF110939 | KF186862 | KF110828 |
| *Prevotella buccae* HJH09 | KF186865 | KF186714 | KF110938 | KF186861 | KF110827 |
| *Prevotella buccae* HJM006 | KF186953 | KF186727 | KF110937 | KF186860 | KF110841 |
| *Prevotella buccae* HJM027 | KF186952 | KF186726 | KF110936 | KF186859 | KF110840 |
| *Prevotella buccae* HJM060 | KF186951 | KF186725 | KF110935 | KF186858 | KF110839 |
| *Prevotella buccae* HJM070 | KF186950 | KF186724 | KF110934 | KF186857 | KF110838 |
| *Prevotella buccae* HJX045 | KF186949 | KF186723 | KF110933 | KF186856 | KF110837 |
| *Prevotella dentalis* HJH26 | KF186948 | KF186687 | KF186864 | KF186843 | KF110836 |
| *Prevotella dentalis* HJM019 | KF186947 | KF186722 | KF110932 | KF186842 | KF110835 |
| *Prevotella dentalis* HJM021 | KF186946 | KF186721 | KF110931 | KF186841 | KF110834 |
| *Prevotella dentalis* HJM035 | KF186930 | KF186708 | KF110918 | KF186840 | KF110977 |
| *Prevotella dentalis* HJM036 | KF186929 | KF186707 | KF110917 | KF186839 | KF110816 |
| *Prevotella dentalis* HJX054 | KF186927 | KF186705 | KF110915 | KF186837 | KF110814 |
| *Prevotella dentalis* HJX055 | KF186926 | KF186704 | KF110914 | KF186836 | KF110826 |
| *Prevotella dentalis* HJX023 | KF186928 | KF186706 | KF110916 | KF186838 | KF110815 |
| *Prevotella dentalis* HJX061 | KF186925 | KF186703 | KF110913 | KF186835 | KF110825 |
| *Prevotella denticola* HJM004 | KF186939 | KF186702 | KF110912 | KF186834 | KF110824 |
| *Prevotella denticola* HJM020 | KF186938 | KF186701 | KF110911 | KF186851 | KF110823 |
| *Prevotella denticola* HJX006 | KF186937 | KF186700 | KF110925 | KF186850 | KF110822 |
| *Prevotella denticola* HJX012 | KF186936 | KF186713 | KF110924 | KF186849 | KF110821 |
| *Prevotella denticola* HJX013 | KF186935 | KF186712 | KF110923 | KF186848 | KF110820 |
| *Prevotella denticola* HJX022 | KF186934 | KF186711 | KF110922 | KF186847 | KF110819 |
| *Prevotella denticola* HJX050 | KF186933 | KF186710 | KF110921 | KF186846 | KF110818 |
| *Prevotella denticola* HJX078 | KF186931 | KF186692 | KF110919 | KF186844 | KF110806 |
| *Prevotella denticola* HJX073 | KF186932 | KF186709 | KF110920 | KF186845 | KF110817 |
| *Prevotella fusca* HJM058 | KF186920 | KF186759 | KF110902 | KF186832 | KF110805 |
| *Prevotella histicola* HJH13 | KF186919 | KF186691 | KF110901 | KF186833 | KF110804 |
| *Prevotella intermedia* HJH14 | KF186886 | KF186690 | KF110900 | KF186819 | KF110803 |
| *Prevotella intermedia* HJH16 | KF186885 | KF186689 | KF110899 | KF186818 | KF110802 |
| *Prevotella intermedia* HJH19 | KF186884 | KF186688 | KF110898 | KF186817 | KF110801 |
| *Prevotella intermedia* HJM010-2 | KF186883 | KF186699 | KF110897 | KF186816 | KF110813 |
| *Prevotella intermedia* HJM018 | KF186882 | KF186698 | KF110896 | KF186815 | KF110812 |
| *Prevotella intermedia* HJM022 | KF186881 | KF186697 | KF110910 | KF186814 | KF110811 |
| *Prevotella intermedia* HJM030 | KF186880 | KF186696 | KF110909 | KF186813 | KF110810 |
| *Prevotella intermedia* HJM034-2 | KF186879 | KF186695 | KF110908 | KF186812 | KF110809 |
| *Prevotella intermedia* HJM055 | KF186878 | KF186694 | KF110907 | KF186811 | KF110808 |
| *Prevotella intermedia* HJM056 | KF186877 | KF186693 | KF110906 | KF186810 | KF110807 |
| *Prevotella intermedia* HJM075 | KF186873 | KF186767 | KF110890 | KF186806 | KF110876 |
| *Prevotella intermedia* HJM083 | KF186872 | KF186766 | KF110889 | KF186805 | KF110875 |
| *Prevotella intermedia* HJM085 | KF186871 | KF186765 | KF110895 | KF186804 | KF110874 |
| *Prevotella intermedia* HJM095 | KF186870 | KF186774 | KF110894 | KF186803 | KF110888 |
| *Prevotella intermedia* HJM096 | KF186869 | KF186773 | KF110893 | KF186802 | KF110887 |
| *Prevotella intermedia* HJX018 | KF186868 | KF186772 | KF110892 | KF186801 | KF110886 |
| *Prevotella intermedia* HJX024 | KF186867 | KF186771 | KF110891 | KF186800 | KF110885 |
| *Prevotella intermedia* HJX028 | KF186866 | KF186756 | KF110968 | KF186799 | KF110884 |
| *Prevotella intermedia* HJX031 | KF186894 | KF186755 | KF110967 | KF186798 | KF110883 |
| *Prevotella intermedia* HJX057 | KF186892 | KF186753 | KF110965 | KF186796 | KF110881 |
| *Prevotella intermedia* HJX058 | KF186891 | KF186752 | KF110964 | KF186824 | KF110880 |
| *Prevotella intermedia* HJX060 | KF186890 | KF186751 | KF110963 | KF186823 | KF110866 |
| *Prevotella intermedia* HJX070 | KF186889 | KF186750 | KF110976 | KF186822 | KF110865 |
| *Prevotella intermedia* HJX080 | KF186888 | KF186764 | KF110975 | KF186821 | KF110864 |
| *Prevotella intermedia* HJM057-2 | KF186876 | KF186770 | KF110905 | KF186809 | KF110879 |
| *Prevotella intermedia* HJM068 | KF186875 | KF186769 | KF110904 | KF186808 | KF110878 |
| *Prevotella intermedia* HJM069 | KF186874 | KF186768 | KF110903 | KF186807 | KF110877 |
| *Prevotella intermedia* HJX081 | KF186887 | KF186763 | KF110974 | KF186820 | KF110863 |
| *Prevotella intermedia* HJX032 | KF186893 | KF186754 | KF110966 | KF186797 | KF110882 |
| *Prevotella melaninogenica* HJH10 | KF186910 | KF186762 | KF110973 | KF186790 | KF110862 |
| *Prevotella melaninogenica* HJH25 | KF186909 | KF186761 | KF110972 | KF186789 | KF110861 |
| *Prevotella melaninogenica* HJX001 | KF186906 | KF186757 | KF110969 | KF186786 | KF110873 |
| *Prevotella melaninogenica* HJX005 | KF186914 | KF186743 | KF110957 | KF186785 | KF110872 |
| *Prevotella melaninogenica* HJX010 | KF186913 | KF186742 | KF110956 | KF186784 | KF110871 |
| *Prevotella melaninogenica* HJX014 | KF186912 | KF186686 | KF110955 | KF186783 | KF110870 |
| *Prevotella melaninogenica* HJX059 | KF186911 | KF186741 | KF110954 | KF186782 | KF110869 |
| *Prevotella melaninogenica* HJM071 | KF186907 | KF186758 | KF110970 | KF186787 | KF110859 |
| *Prevotella melaninogenica* HJM037 | KF186908 | KF186760 | KF110971 | KF186788 | KF110860 |
| *Prevotella multiformis* HJH29 | KF186918 | KF186740 | KF110953 | KF186781 | KF110868 |
| *Prevotella nigrescens* HJM003 | KF186898 | KF186749 | KF110951 | KF186779 | KF110851 |
| *Prevotella nigrescens* HJM025 | KF186897 | KF186748 | KF110950 | KF186778 | KF110850 |
| *Prevotella nigrescens* HJM032 | KF186896 | KF186747 | KF110949 | KF186777 | KF110849 |
| *Prevotella nigrescens* HJM053 | KF186895 | KF186746 | KF110962 | KF186776 | KF110848 |
| *Prevotella nigrescens* HJM066 | KF186905 | KF186745 | KF110961 | KF186775 | KF110847 |
| *Prevotella nigrescens* HJX002 | KF186904 | KF186744 | KF110960 | KF186795 | KF110846 |
| *Prevotella nigrescens* HJX033 | KF186902 | KF186731 | KF110958 | KF186793 | KF110858 |
| *Prevotella nigrescens* HJX052 | KF186900 | KF186729 | KF110941 | KF186791 | KF110856 |
| *Prevotella nigrescens* HJX046 | KF186901 | KF186730 | KF110942 | KF186792 | KF110857 |
| *Prevotella nigrescens* HJH18 | KF186899 | KF186739 | KF110952 | KF186780 | KF110867 |
| *Prevotella nigrescens* HJX026 | KF186903 | KF186732 | KF110959 | KF186794 | KF110845 |
| *Prevotella oris* HJH23 | KF186917 | KF186728 | KF110940 | KF186827 | KF110855 |
| *Prevotella oris* HJX004 | KF186916 | KF186738 | KF110948 | KF186826 | KF110854 |
| *Prevotella oulorum* HJM031 | KF186915 | KF186737 | KF110947 | KF186825 | KF110853 |
| *Prevotella pallens* HJM028 | KF186924 | KF186736 | KF110946 | KF186831 | KF110852 |
| *Prevotella pallens* HJM061 | KF186923 | KF186735 | KF110945 | KF186830 | KF110842 |
| *Prevotella salivae* HJX069 | KF186922 | KF186734 | KF110944 | KF186829 | KF110844 |
| *Prevotella veroralis* HJX021 | KF186921 | KF186733 | KF110943 | KF186828 | KF110843 |
